# Comparing deep learning and concept extraction based methods for patient phenotyping from clinical narratives

**DOI:** 10.1371/journal.pone.0192360

**Published:** 2018-02-15

**Authors:** Sebastian Gehrmann, Franck Dernoncourt, Yeran Li, Eric T. Carlson, Joy T. Wu, Jonathan Welt, John Foote, Edward T. Moseley, David W. Grant, Patrick D. Tyler, Leo A. Celi

**Affiliations:** 1 MIT Critical Data, Laboratory for Computational Physiology, Cambridge, MA, United States of America; 2 Harvard SEAS, Harvard University, Cambridge, MA, United States of America; 3 Massachusetts Institute of Technology, Cambridge, MA, United States of America; 4 Adobe Research, San Jose, CA, United States of America; 5 Harvard T.H. Chan School of Public Health, Cambridge, MA, United States of America; 6 Philips Research North America, Cambridge, MA, United States of America; 7 Wellman Center for Photomedicine, Massachusetts General Hospital, Boston, MA, United States of America; 8 Tufts University School of Medicine, Cambridge, MA, United States of America; 9 College of Science and Mathematics, University of Massachusetts, Boston, MA, United States of America; 10 Department of Surgery, Division of Plastic and Reconstructive Surgery, Washington University School of Medicine, St. Louis, MO, United States of America; 11 Department of Internal Medicine, Beth Israel Deaconess Medical Center, Boston, MA, United States of America; Centers for Disease Control, TAIWAN

## Abstract

In secondary analysis of electronic health records, a crucial task consists in correctly identifying the patient cohort under investigation. In many cases, the most valuable and relevant information for an accurate classification of medical conditions exist only in clinical narratives. Therefore, it is necessary to use natural language processing (NLP) techniques to extract and evaluate these narratives. The most commonly used approach to this problem relies on extracting a number of clinician-defined medical concepts from text and using machine learning techniques to identify whether a particular patient has a certain condition. However, recent advances in deep learning and NLP enable models to learn a rich representation of (medical) language. Convolutional neural networks (CNN) for text classification can augment the existing techniques by leveraging the representation of language to learn which phrases in a text are relevant for a given medical condition. In this work, we compare concept extraction based methods with CNNs and other commonly used models in NLP in ten phenotyping tasks using 1,610 discharge summaries from the MIMIC-III database. We show that CNNs outperform concept extraction based methods in almost all of the tasks, with an improvement in F1-score of up to 26 and up to 7 percentage points in area under the ROC curve (AUC). We additionally assess the interpretability of both approaches by presenting and evaluating methods that calculate and extract the most salient phrases for a prediction. The results indicate that CNNs are a valid alternative to existing approaches in patient phenotyping and cohort identification, and should be further investigated. Moreover, the deep learning approach presented in this paper can be used to assist clinicians during chart review or support the extraction of billing codes from text by identifying and highlighting relevant phrases for various medical conditions.

## Introduction

The secondary analysis of data from electronic health records (EHRs) is crucial to better understand the heterogeneity of treatment effects and to individualize patient care [1]. With the growing adoption rate of EHRs [2], researchers gain access to rich data sets, such as the Medical Information Mart for Intensive Care (MIMIC) database [3, 4], and the Informatics for Integrating Biology and the Bedside (i2b2) datamarts [5–10]. These data sets can be explored and mined in numerous ways [11]. EHR data comprise both structured data such as International Classification of Diseases (ICD) codes, laboratory results and medications, and unstructured data such as clinician progress notes. While structured data do not require complex processing prior to statistical tests and machine learning tasks, the majority of data exist in unstructured form [12]. Natural language processing methods can extract this valuable data, which in conjunction with analyzing structured data can lead to a better understanding of health and diseases [13] and to a more accurate phenotyping of patients to compare tests and treatments [14–16]. Patient phenotyping is a classification task for determining whether a patient has a medical condition or for pinpointing patients who are at risk for developing one. Further, intelligent applications for patient phenotyping can support clinicians by reducing the time they spend on chart reviews, which takes up a significant fraction of their daily workflow [17, 18].

A popular approach to patient phenotyping using NLP is based on extracting medical phrases from texts and using them as input to build a predictive model [19]. The dictionary of relevant phrases is often task-specific and its development requires significant effort and a deep understanding of the task from domain experts [20]. A different approach is to develop a fully rule-based algorithm for each condition [21]. Due to the laborious task required of clinicians to build a generalizable model for patient phenotyping, models for automated classification using NLP are rarely developed outside of the research area. However, utilizing recent developments in deep learning for phenotyping might prove to be a generalizable approach with less intense domain expert involvement. Applications of deep learning for other tasks in healthcare have shown promising results; examples include mortality prediction [22], patient note de-identification [23], skin cancer detection [24], and diabetic retinopathy detection [25].

A possible drawback to deep learning models is their lack of interpretability. Interpretability means how easily one can understand how a model arrived at a prediction [26]. This is crucial for healthcare applications since results can directly impact decisions about patients health. Furthermore, clinicians have intimate pre-existing knowledge and thus expect applications to support their decision making as opposed to make decisions for them. Therefore, interpretable models are required so that clinicians can trust and control their results [27]. Moreover, the European Union is considering regulations that require algorithms to be interpretable [28]. While much work has been done to understand deep learning NLP models and develop understandable models [29–31], their complex interactions between inputs are inherently less interpretable than linear models that use predefined phrase dictionaries.

In this work, we assess convolutional neural networks (CNNs) as an approach to text-based patient phenotyping. CNNs are designed to identify phrases in text that lead to a positive or negative classification, similar to the phrase dictionary approach, and outperform approaches to classification problems in other domains [32–34]. We compare CNNs to entity extraction systems using the Mayo clinical Text Analysis and Knowledge Extraction System (cTAKES) [35], and other NLP methods such as logistic regression models using n-gram features. Using a corpus of 1,610 discharge summaries that were annotated for ten different phenotypes, we show that CNNs outperform both extraction-based and n-gram-based methods. Finally, we evaluate the interpretability of the model by assessing the learned phrases that are associated with each phenotype and compare them to the phrase dictionaries developed by clinicians.

## Background

Accurate patient phenotyping is required for secondary analysis of EHRs to correctly identify the patient cohort and to better identify the clinical context [36, 37]. Studies employing a manual chart review process for patient phenotyping are naturally limited to a small number of preselected patients. Therefore, NLP is necessary to identify information that is contained in text but may be inconsistently captured in the structured data, such as recurrence in cancer [20, 38], whether a patient smokes [5], classification within the autism spectrum [39], or drug treatment patterns [40]. However, unstructured data in EHRs, for example progress notes or discharge summaries, are not typically amenable to simple text searches because of spelling mistakes, synonyms, and ambiguous terms [41]. To help address these issues, researchers utilize dictionaries and ontologies for medical terminologies such as the unified medical language system (UMLS) [42] and the systematized nomenclature of medicine—clinical terms (SNOMED CT) [43].

Examples of systems that employ such databases and extract concepts from text are the KnowledgeMap Concept Identifier (KMCI) [44], MetaMap [45], Medlee [46], MedEx [47], and the cTAKES. These systems identify phrases within a text that correspond to medical entities [35, 48]. This significantly reduces the work required from researchers, who previously had to develop task-specific extractors [49]. Extracted entities are typically filtered to only include concepts related to the patient phenotype under investigation and either used as features for a model that predicts whether the patient fits the phenotype, or as input for rule-based algorithms [19, 39, 50]. Liao et al. [13] describe the process of extraction, rule-generation and prediction as the general approach to patient phenotyping using the cTAKES [14, 51–53], and test this approach on various data sets [54]. The role of clinicians in this task is both to annotate data and to develop a task-specific dictionary of phrases that are relevant to a patient phenotype. Improving existing approaches often requires significant additional time-investment from the clinicians, for example by developing and combining two separate phrase-dictionaries for pathology documents and clinical documents [20]. The cost and time required to develop these algorithms limit their applicability to large or repeated tasks. While a usable system would offset the development costs, it does not address the problem that a specialized NLP system would have to be developed for every task in a hospital. Moreover, algorithms often do not transfer well between different hospitals, warranting extensive transferability studies [55]. The deep learning based approach we evaluate in this paper does not require any hand-crafted input and can easily be retrained with new data. This can potentially increase the transferability of studies while removing the time required to develop new phrase-dictionaries.

## Materials and methods

### Data

The notes used in this study are extracted from the MIMIC-III database. MIMIC-III contains de-identified clinical data of over 53,000 hospital admissions for adult patients to the intensive care units (ICU) at the Beth Israel Deaconess Medical Center from 2001 to 2012. The dataset comprises several types of clinical notes, including discharge summaries (n = 52,746) and nursing notes (n = 812,128). We focus on the discharge summaries since they are the most informative for patient phenotyping [56]. More specifically, we investigate phenotypes that may associate a patient with being a ‘frequent flyer’ in the ICU (defined as >= 3 ICU visits within 365 days). As many as one third of readmissions have been suggested to be preventable; identifying modifiable risk factors is a crucial step to reducing them [57]. We extracted the discharge summary of the first visit from 415 ICU frequent flyers in MIMIC-III, as well as 313 randomly selected summaries from later visits of the same patients. We additionally selected 882 random summaries from patients who are not frequent flyers, yielding a total of 1,610 notes. The cTAKES output for these notes contains a total of 11,094 unique CUIs.

All 1,610 notes were annotated for the ten phenotypes described in Table 1. The table shows the definitions for each investigated phenotype. To ensure high-quality labels and minimize errors, each note was labeled at least twice for each phenotype. Annotators include two clinical researchers (ETM, JW), two junior medical residents (JF, JTW), two senior medical residents (DWG, PDT), and a practicing intensive care medicine physician (LAC). In the case that the annotators were unsure, one of the senior clinicians (DWG or PDT) decided on the final label. The table further shows the number of occurrences of each phenotype as a measure of dataset imbalance. The frequency varies from 126 to 460 cases, which corresponds to between 7.5% and 28.6% of the dataset. Finally, we provide the Cohen’s Kappa measure for inter-rater agreement. While well specified phenotypes such as depression have a very high agreement (0.95), other phenotypes, such as chronic neurologic dystrophies, have a lower agreement 0.71 and required more interventions by senior clinicians.

**Table 1:** The ten different phenotypes used for this study. The first column shows the name of the phenotype, the second column shows the number of positive examples our of the total 1,610 notes, and the third shows the *κ* coefficient as inter-rater agreement measure. The last column lists the definition for each phenotype that was used to identify and annotate the phenotype.

Phenotype]	#pos.	*κ*	Definition
Adv. / Metastatic Cancer	161	0.83	Cancers with very high or imminent mortality (pancreas, esophagus, stomach, cholangiocarcinoma, brain); mention of distant or multi-organ metastasis, where palliative care would be considered (prognosis < 6 months).
Adv. Heart Disease	275	0.82	Any consideration for needing a heart transplant; description of severe aortic stenosis (aortic valve area < 1.0cm^2^), severe cardiomyopathy, Left Ventricular Ejection Fraction (LVEF) <= 30%. Not sufficient to have a medical history of congestive heart failure (CHF) or myocardial infarction (MI) with stent or coronary artery bypass graft (CABG) as these are too common.
Adv. Lung Disease	167	0.81	Severe chronic obstructive pulmonary disease (COPD) defined as Gold Stage III-IV, or with a forced expiratory volume during first breath (FEV1) < 50% of normal, or forced vital capacity (FVC) < 70%, or severe interstitial lung disease (ILD), or Idiopathic pulmonary fibrosis (IPF).
Chronic Neurologic Dystrophies	368	0.71	Any chronic central nervous system (CNS) or spinal cord diseases, included/not limited to: Multiple sclerosis (MS), amyotrophic lateral sclerosis (ALS), myasthenia gravis, Parkinson’s Disease, epilepsy, history of stroke/cerebrovascular accident (CVA) with residual deficits, and various neuromuscular diseases/dystrophies.
Chronic Pain	321	0.83	Any etiology of chronic pain, including fibromyalgia, requiring long-term opioid/narcotic analgesic medication to control.
Alcohol Abuse	196	0.86	Current/recent alcohol abuse history; still an active problem at time of admission (may or may not be the cause of it).
Substance Abuse	155	0.86	Include any intravenous drug abuse (IVDU), accidental overdose of psychoactive or narcotic medications,(prescribed or not). Admitting to marijuana use in history is not sufficient.
Obesity	126	0.94	Clinical obesity. BMI > 30. Previous history of or being considered for gastric bypass. Insufficient to have abdominal obesity mentioned in physical exam.
Psychiatric disorders	295	0.91	All psychiatric disorders in DSM-5 classification, including schizophrenia, bipolar and anxiety disorders, other than depression.
Depression	460	0.95	Diagnosis of depression; prescription of anti-depressant medication; or any description of intentional drug overdose, suicide or self-harm attempts.

### Concept-extraction based methods

We use cTAKES to extract concepts from each note. In cTAKES, sentences and phases are first split into tokens (individual words). Then, tokens with variations (e.g. plural) are normalized to their base form. The normalized tokens are tagged for their part-of-speech (e.g. noun, verb), and a shallow parse tree is constructed to represent the grammatical structure of a sentence. Finally, a named-entity recognition algorithm uses this information to detect named entities for which a concept unique identifier (CUI) exists in UMLS [43].

Related approaches then use relevant concepts in a note as input to machine learning algorithms to directly learn to predict a phenotype [58, 59]. We specify two different approaches to using the cTAKES output. The first approach uses the complete list of extracted CUIs as input to further processing steps. In the second approach, clinicians specify a dictionary comprising all clinical concepts that are relevant to the desired phenotype (e.g. Alcohol Abuse) as described by Carrell et al. [20].

Our predictive models replicate the process as described by Liao et al. [13]. Each note is represented as a bag-of-CUIs by counting the number of occurrences of each of the CUIs. Due to the fact that cTAKES detects negations, occurrences of negated and non-negated CUIs are counted separately. These features are then transformed using the term frequency–inverse document frequency (TF-IDF). Compared to the bag-of-CUIs, or the bag-of-words of a note as described by Halpern et al. [16], the TF-IDF of the features reflects the importance of a feature to a note. For an accurate comparison to approaches in literature, we train a random forest (RF), a naive Bayes (NB), and a logistic regression (LR) model with these features.

### Convolutional neural networks

**Fig 1:**
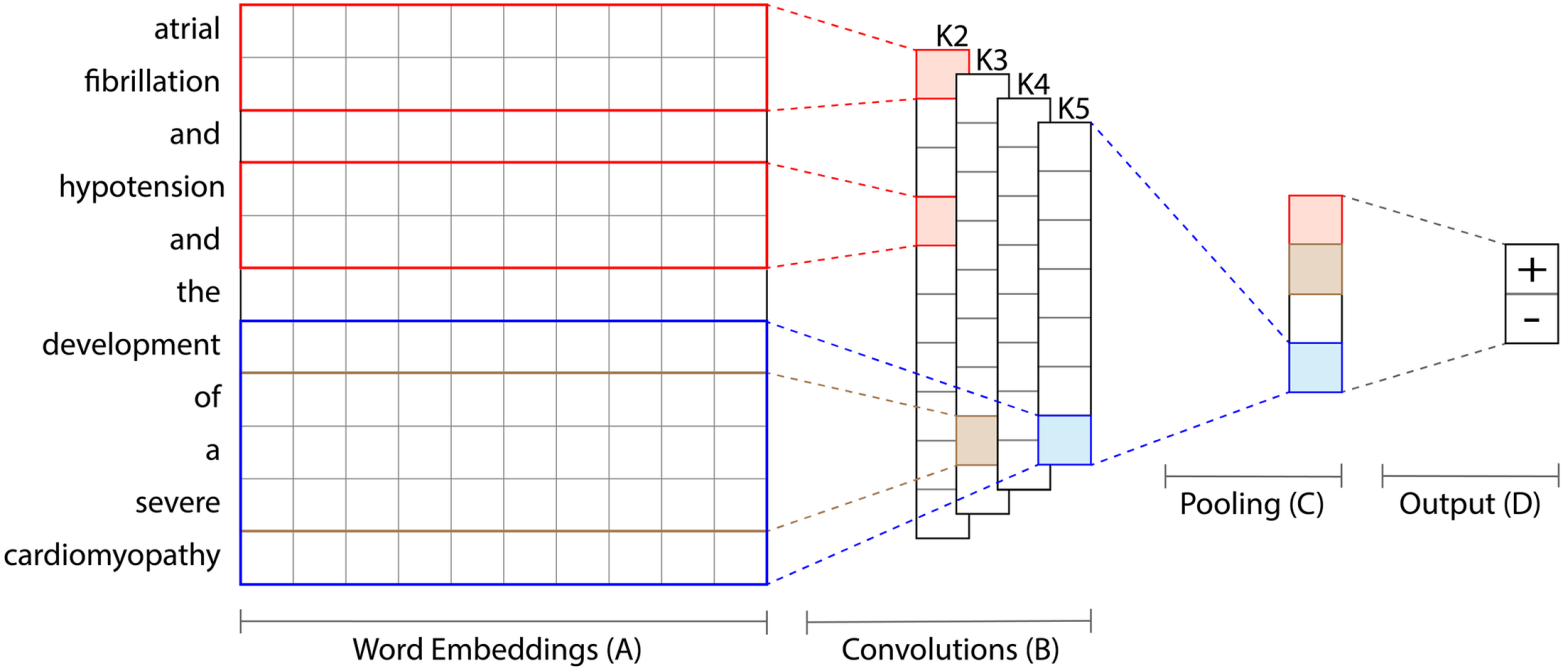
Overview of the basic CNN architecture. (A) Each word within a discharge note is represented as its word embedding. In this example, both instances of the word “and” will have the same embedding. (B) Convolutions of different widths are used to learn filters that are applied to word sequences of the corresponding length. The convolution K2 with width 2 in the example looks at all 10 combinations of neighboring two words and output one value each. There can be multiple feature maps for each convolution width. (C) The multiple resulting vectors are reduced to only the highest value (the one with the most signaling power) for each of the different convolutions. (D) The final prediction (“Does the phenotype apply to the patient?”) is made by computing a weighted combination of the pooled values and applying a sigmoid function, similar to a logistic regression. This figure is adapted with permission from Kim [33].

We use a convolutional neural network (CNN) for text classification to represent deep learning methods, replicating the architecture proposed by Collobert et al. and Kim [33, 60]. The idea behind convolutions in computer vision is to learn filters that transform adjacent pixels into single values [61]. Equivalently, a CNN for NLP learns which combinations of adjacent words are associated with a given concept. An overview of our architecture is shown in Fig 1.

In a CNN, a text is first represented as a sequence of word embeddings in which each word is projected into a distributed representation. A word $x_{i}\in R^{k}$ is the *k*-dimensional embedding vector for the *i*-th word in a text. Consequently, a text of length *n* is represented the concatenation of its word embeddings **x**_1:*n*_ = **x**_1_ ⊕ **x**_2_ ⊕ … ⊕ **x**_*n*_ (where ⊕ is the concatenation operation). Word embeddings have shown to improve performance on other tasks based on EHRs, for example named-entity recognition [62]. Words that occur in similar contexts are trained to have similar word embeddings. Therefore, misspellings, synonyms and abbreviations of an original word learn similar embeddings, which lead to similar results. Consequently, a database of synonyms and common misspellings is not required [20]. Word embeddings can be pre-trained on a larger corpus of texts, which improves results of the NLP system and reduces the amount of data required to train a model [63, 64]. We pre-train our embeddings with word2vec [65] on all discharge notes available in the MIMIC-III database [4].

The embedded text is used as input to the convolutional layer. Convolutions detect a signal from a combination of adjacent inputs. Each convolutional operation applies a filter of trained parameters $w\in R^{hk}$ to an input-window of width *h*. A resulting feature *c*_*i*_ is computed as *c*_*i*_ = *f*(**w** ⋅ **x**_*i*:*i*+*h*−1_ + *b*). In this equation, b∈R represents a bias term, and *f* a non-linear function, in our case a rectified linear unit f(x)=max(0,x). A filter is applied to every possible word window in the input to produce a feature map **c** = [*c*_1_, *c*_2_, …, *c*_*n*−*h*+1_]. This feature map is then reduced to a single value using a pooling operation. More specifically, we use max-over-time-pooling to extract the most predictive value $\hat{c}=\text{max}\left(c\right)$ [60]. We combine multiple convolutions per length and of different lengths to evaluate phrases from one to five words long, as illustrated in Fig 1. All convolutions use the same word embeddings as input and only differ in the filters they learn. The combination of many filters of varying length results in multiple outputs $z=[{\hat{c}}_{1},\ldots ,{\hat{c}}_{m}]$. A final probability whether the text refers to a patient with a certain condition is computed as *y* = *σ*(**w** ⋅ **z** + *b*), with two trainable parameters **w** and *b* and the sigmoid function *σ*. We train a separate CNN for each phenotype to minimize the negative conditional log-likelihood of training data $L\left(\theta \right)=-\sum_{i=1}^{n}\log p\left(y_{i}|x_{i};\theta \right)$ for a set of parameters *θ*.

### Other baselines

We further investigate a number of baselines to compare with the more complex approaches. We start with a bag-of-words based logistic regression and gradually increase the complexity of the baselines until we reach the CNN. This provides an overview of how adding more complex features impact the performance in this task. Moreover, this investigation shows what factors contribute most to the CNN performance.

#### Bag of words

The simplest possible representation for a note is a bag of words (BoW) which counts phrases of length 1. Let F denote the vocabulary of all words, and *f*_*i*_ the word at position *i*. Let further $\delta \left(f_{i}\right)\in R^{1\times |F|}$ be a one-hot vector with a one at position *f*_*i*_. Then, a note **x** is represented as **x** = ∑_*i*_ *δ*(*f*_*i*_). A prediction is made by computing *y* = *σ*(**w** ⋅ **x** + *b*), where **w** and *b* are trainable parameters.

#### n-grams

The bag-of-words approach can be extended to include representations for longer phrases, also called n-grams, as well. Consider a note “The sick patient”. While a bag-of-words approach considers each word separately, a two-gram model additionally considers all possible phrases of length two. Thus, the model would also consider the phrases “The sick” and “sick patient”. Since the number of possible phrases grows exponentially in the size of the vocabulary, the data for longer phrases becomes very sparse and the n-gram size can’t be increased too much. In this study, we show results for models with phrase lengths of up to 5.

#### Embedding-based logistic regression

A typical CNN differs from n-gram based models in both feature representation and model architecture. To study whether models simpler than a CNN have the same expressive power with the same feature representation, we modify the LR to use the same word embeddings as the CNN. Here, a note is represented as a sequence of its word embeddings **x**_1:*n*_ = **x**_1_ ⊕ **x**_2_ ⊕ … ⊕ **x**_*n*_. However, using this representation as input to a LR means that every word is sensitive to its location in a text. An occurrence of “heart” at position 5 would use different parameters in *w* than the same word at position 10. Therefore, we change the model to use the same weight vector *w* for each word embedding in a text, computing the score as $\sum_{i=1}^{n}w\cdot x_{i}$. The representation also poses the challenge that inputs vary in length. Thus, we normalize the score by dividing by the length *n*. Unfortunately, the capacity of this adjusted logistic regression model is insufficient. The AUC of this model is close to 0.5 for all phenotypes, which means that it is not better than chance. Since a typical note can be longer than 4,000 words, all the expressive terms are smoothed out. Using max-pooling instead of averaging as an approach to mitigating this problem has the same result. We thus omit this baseline from further results.

#### CNN without convolutions

In order to account for the problem that relevant features are smoothed out by the number of features in the model, we train several weight parameters **w** instead of only one and combine the pooled results by concatenating them. Then, we take the resulting vector as input to another logistic regression. This architecture is equivalent to a CNN with a convolutional width of one, for which we show results in S1 Table.

### Interpretability

The inability of humans to understand predictions of complex machine learning models poses a difficult challenge when such approaches are used in healthcare [26]. Therefore, we consider it crucial that well-performing approaches should be understood and trusted by those who use them. Moreover, bias in the sources of data could lead the model to learn false implications. One such example of bias was in mortality prediction among patients with pneumonia where asthma was found to increase survival probability [27, 66]. This result was due to an institutional practice of admitting all patients with pneumonia and a history of asthma to the ICU regardless of disease severity, so that a history of asthma was strongly correlated with a lower illness severity. To measure the interpretability of each approach, we consider the most frequently used approach in text-classification. In this approach, users are shown a list of phrases or concepts that are most salient for a particular prediction; this list either manifests as an actual list or as highlights of the original text [67, 68].

As a baseline we consider the filtered cTAKES random forest approach (other baselines can be treated equivalently). In the filtered cTAKES approach, clinicians ensure that all features directly pertain to the specific phenotype [22]. We rank the importance of each remaining CUI using the gini importance of the trained model [69]. The resulting ranking is a direct indication of the globally most relevant CUIs. An individual document can be analyzed by ranking only the importance of CUIs that occur in this document.

For the CNN, we propose a modified version of the saliency as defined by Li et al. to compute the most relevant phrases [70]. They define the saliency for a neural network as the norm of the gradient of the Loss function for a particular prediction with respect to an input *x*_*i*_ as

$$\begin{array}{c} S_{1}\left(x_{i}\right)=\left|\frac{\partial L(1,\hat{y}}{\partial x_{i}}\right| \end{array}$$

In the CNN case, an input is only a single word or a single dimension of its embedding. Thus, we extend the saliency definition to instead compute the gradient with respect to the learned feature maps, which we call phrase-saliency. This approximates how much a phrase contributed to a prediction instead of a single word.

$$\begin{array}{c} S_{1}\left(x_{i:i+h-1}\right)=\left|\frac{\partial L(1,\hat{y}}{\partial c_{i}}\right| \end{array}$$

Since we employ multiple feature maps of different widths, we compute the phrase-saliency across all of them, and use those with maximum value. The saliency is measured on a per-document basis. To arrive at the globally most relevant phrases, we iterate over all documents and measure the phrases that had the highest phrase-saliency while removing duplicate phrases. Alternative methods not considered in this work search the local space around an input [71], or compute a layer-wise backpropagation [72–75].

### Evaluation-quantitative performance measures

We evaluate the precision, recall, F1-score, and area under the ROC curve (AUC) of all models as a quantitative measure. The F-score is derived from the confusion matrix for the results on the test set. A confusion matrix contains four counts: true positive (TP), false positive (FP), true negative (TN), and false negative (FN). The precision *P* is the fraction of correct predictions out of all the samples that were predicted to be positive $\frac{\text{TP}}{\text{TP}+\text{FP}}$. The recall *R* is the percentage of true positive predictions in relation to all the predictions that should have been predicted as positive $\frac{\text{TP}}{\text{TP}+\text{FN}}$. The F1-score is the harmonic mean of both precision and recall $2*\frac{P*R}{P+R}$.

For all models, the data is randomly split into a training, validation, and test set. 70% of the labeled data is used as the training set, 10% as validation set and 20% as test set. While splitting, we ensure that patients’ notes stay within the set, so that all discharge notes in the test set are from patients not previously seen by the model. The reported numbers across different models for the same phenotype are obtained from testing on the same test set. The validation set is used to choose the hyperparameters for the models. A detailed description of hyperparameters is shown in S1 Text.

All models are trained separately for each of the phenotypes to examine the results in isolation. We present the results for model configurations with the highest F1-score for each model in the main part of the paper. Additional results for different convolution widths for the CNN are shown in S1 Table, and for different models using cTAKES in S2 Table For the other baselines, we present the results of a bag of words representation, and the best performing n-gram length, and additional results in S3 Table.

In summary, we present results for the following approaches:

**CNN** The convolutional neural network with best performing convolution width
**BoW** Baseline using a bag of words representation of a note and logistic regression
**n-gram** Baseline using an n-gram representation of a note and logistic regression
**cTAKES full** The best performing model that uses the full output from cTAKES
**cTAKES filter** The best performing model using the filtered CUI-list from cTAKES

### Assessing interpretability

In order to evaluate how understandable the predictions of the different approaches are, we conducted a study of the globally most relevant phrases and CUIs. For each phenotype, we computed the five most relevant features, yielding a total of 50 phrases and 50 CUIs. We then asked clinicians to rate the features on a scale from 0 to 3 with the following descriptions for each rating:

**0** The phrase/CUI is unrelated to the phenotype.
**1** The phrase is associated with the concept subjectively from clinical experience, but is not directly related (e.g. alcohol abuse for psychiatric disorder).
**2** The phrase has to do with the concept, but is not a definite indicator of its existence (e.g. a medication).
**3** The phrase is a direct indicator of the concept or very relevant (e.g. history of COPD for advanced lung disease).

The features were shown without context other than the name of the phenotype. We additionally provided an option to enter free text comments for each phenotype. We note that all participating clinicians were involved with annotation of the notes and are aware of the definitions for the phenotypes. They were not told about the origin of the phrases before rating them in order to prevent potential bias. In total, we collected 300 ratings, an average of three per feature.

## Results

**Fig 2:**
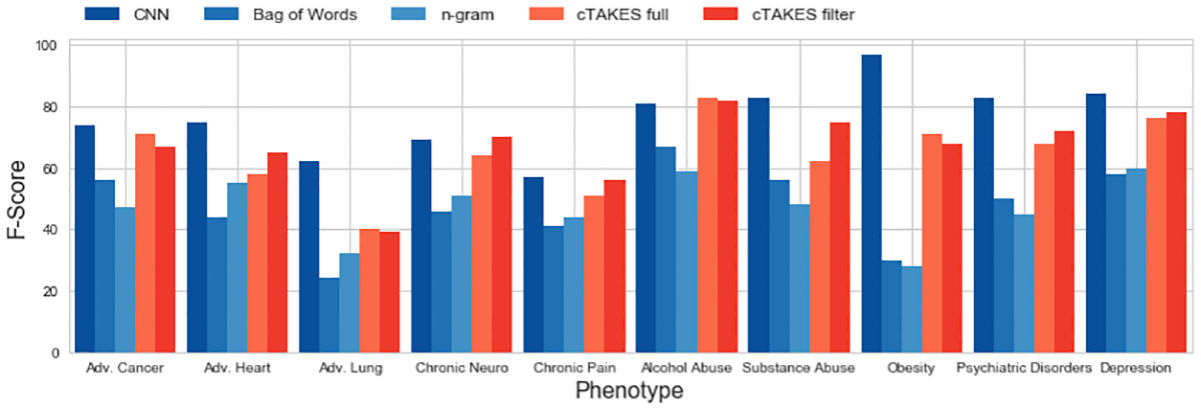
Comparison of achieved F1-scores across all tested phenotypes. The left three models directly classify from text, the right two models are concept-extraction based. The CNN outperforms the other models on most tasks.

We show an overview of the F1-scores for different models and phenotypes in Fig 2. For almost all phenotypes, the CNN outperforms all other approaches. For some of the phenotypes such as Obesity and Psychiatric Disorders, the CNN outperforms the other models by a large margin. A *χ*^2^ test confirms that the CNN’s improvements over both the filtered and the full cTAKES models are statistically significant at a 0.01 level. There is only a minimal improvement when using the filtered cTAKES model, which requires much more effort from clinicians, over the full cTAKES model. The *χ*^2^ test confirms that there is no statistically significant improvement of this method on our data with a p-value of 0.86. We also note that the TF-IDF transformation of the CUIs yielded a small average improvement in AUC of 0.02 (*σ* = 0.03) over all the considered models.

**Fig 3:**
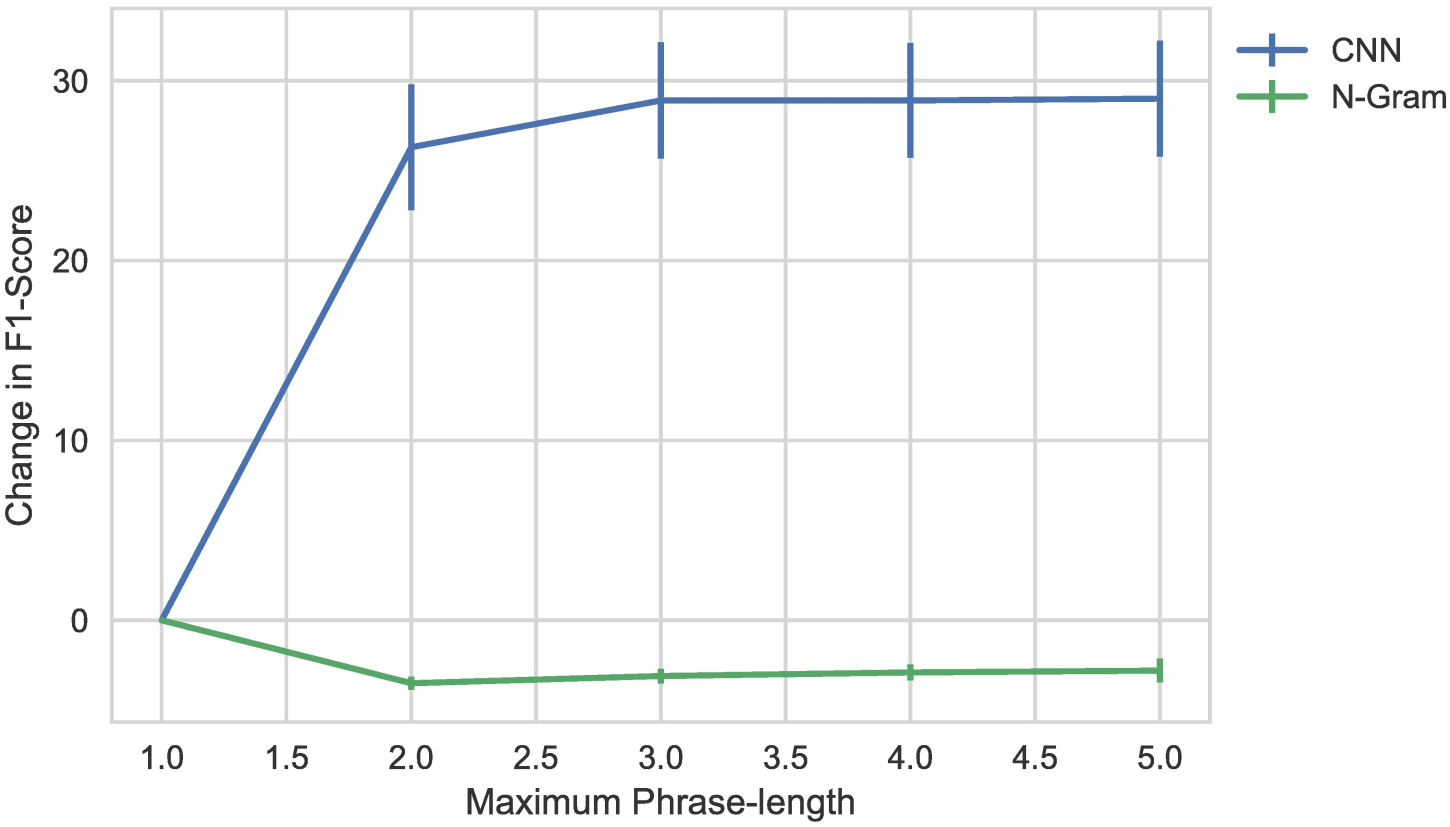
Impact of phrase length on model performance. The figure shows the change in F1-score between a model that considers only single words and a model that phrases up to a length of 5.

In the detailed results, shown in Table 2, we observe that the CNN has the best performance on almost all of the evaluated values. The n-gram and bag-of-words based methods are consistently weaker than the CNN, corroborating the findings in literature that word embeddings improve performance of clinical NLP tasks [62]. We additionally investigate whether considering longer phrases improves model performance. In Fig 3, we show the difference in F1-score between models with phrases up to a certain length and models that use bag-of-words or bag-of-embeddings. The data used for this figure is shown in S1 and S3 Tables. There is no significant difference in performance for longer phrases in n-gram models. There is, however, a significant improvement for phrases longer than one word for the CNN, showing that the CNN model architecture complements the embedding-based approach and contributes to the result of the model.

**Table 2:** This table shows the best performing model for each approach and phenotype. We show precision, recall, F1-Score, and AUC.

		CNN	BoW	n-gram	cTAKES full	cTAKES filter
Adv. Cancer	*P*	**87**	44	41	80	85
	*R*	65	**77**	55	65	55
	*F*1	**74**	56	47	71	67
	*AUC*	**95**	90	88	94	92
Adv. Heart Disease	*P*	74	70	**78**	71	73
	*R*	**76**	32	42	49	59
	*F*1	**75**	44	55	58	65
	*AUC*	**91**	85	85	88	89
Adv. Lung Disease	*P*	**67**	21	27	**67**	43
	*R*	**57**	29	39	29	36
	*F*1	**62**	24	32	40	39
	*AUC*	**89**	76	79	81	87
Chronic Neuro	*P*	69	47	49	75	**80**
	*R*	**70**	46	54	55	62
	*F*1	69	46	51	64	**70**
	*AUC*	84	72	71	87	**86**
Chronic Pain	*P*	**78**	33	42	66	66
	*R*	45	54	46	41	**48**
	*F*1	**57**	41	44	51	56
	*AUC*	73	68	67	78	**85**
Alcohol Abuse	*P*	85	**100**	55	88	91
	*R*	**79**	50	64	**79**	75
	*F*1	81	67	59	**83**	82
	*AUC*	**96**	89	88	95	**96**
Substance Abuse	*P*	83	62	83	**93**	87
	*R*	**83**	50	33	47	67
	*F*1	**83**	56	48	62	75
	*AUC*	**97**	90	86	**97**	**97**
Obesity	*P*	**100**	27	44	64	62
	*R*	**95**	35	20	80	75
	*F*1	**97**	30	28	71	68
	*AUC*	**100**	72	71	99	98
Psychiatric Disorders	*P*	**87**	47	53	74	81
	*R*	**80**	53	39	63	64
	*F*1	**83**	50	45	68	72
	*AUC*	**95**	77	76	88	93
Depression	*P*	**90**	51	51	81	79
	*R*	**79**	67	73	72	77
	*F*1	**84**	58	60	76	78
	*AUC*	93	77	78	**94**	91

Experiments that used both raw text and CUIs as input to a CNN showed no improvement over only using the text as input. This shows that the information encoded in the CUIs is already available in the text and is detected by the CNN. We hypothesize that encoding information available in UMLS beyond the CUI itself can help to improve the phenotype detection in future work.

We show the most salient phrases according to the CNN and the filtered cTAKES LR models for Advanced Heart Disease and for Alcohol Abuse in Table 3. Both tables contain many of the phrases mentioned in the definition shown in Table 1, such as “Cardiomyopathy”. We also observe mentions of “CHF” and “CABG” for Advanced Heart Disease for both models, which are common medical conditions associated with advanced heart disease, but are not sufficient requirements according to the annotation scheme. The model still learned to associate those phrases with advanced heart disease, since those phrases also occur in many notes from patients that were labeled positive for advanced heart failure. The phrases for Alcohol Abuse illustrate how the CNN can detect mentions of the condition in many forms. Without human input, the CNN learned that EtOH and alcohol are used synonymously with different spellings and thus detects phrases containing either of them. The filtered cTAKES RF model surprisingly ranks victim of abuse higher than the direct mention of alcohol abuse in a note, and finds that it is very indicative of Alcohol Abuse if an ethanol measurement was taken. While the CUIs extracted by cTAKES can be very generic, such as “Atrium, Heart” or “Heart”, the salient CNN phrases are more specific.

**Table 3:** The most salient phrases for advanced heart failure and alcohol abuse. The salient cTAKES CUIs are extracted from the filtered RF model.

cTAKES	CNN
**Advanced Heart Disease**	
Magnesium	Wall Hypokinesis
Cardiomyopathy	Port pacer
Hypokinesia	Ventricular hypokinesis
Heart Failure	p AVR
Acetylsalicylic Acid	post ICD
Atrium, Heart	status post ICD
Coronary Disease	EF 20 30
Atrial Fibrillation	bifurcation aneurysm clipping
Coronary Artery	CHF with EF
Disease	cardiomyopathy, EF 15
Aortocoronary Bypasses	(EF 20 30
Fibrillation	coronary artery bypass graft
Heart	respiratory viral infection by DFA
Catheterization	severe global free wall hypokinesis
Chest	Class II, EF 20
Artery	lateral CHF with EF 30
CAT Scans, X-Ray	anterior and atypical hypokinesis akinesis
Hypertension	severe global left ventricular hypokinesis
Creatinine Measurement	’s cardiomyopathy, EF 15
**Alcohol Abuse**	
Victim of abuse	Consciousness Alert
Ethanol Measurement	Alcohol Abuse
Alcohol Abuse	EtOH abuse
Thiamine	Alcoholic Dilated
Social and personal history	ETOH cirrhosis
Family history	heavy alcohol abuse
Hypertension	evening Alcohol abuse
Injuries risk	Drug Reactions Attending
Pain	alcohol withdrawal compartment syndrome
Sodium	EtOH abuse with multiple
Potassium Measurement	liver secondary to alcohol abuse
Plasma Glucose Measurement	abuse crack cocaine, EtOH

In the quantitative study of relevant phrases and CUIs, phrases from the CNN received an average rating of 2.44 (*σ* = 0.89), and the cTAKES based approach received an average rating of 1.9 (*σ* = 0.97). A t-test for two independent samples showed that there is a statistically significant difference between the two with a p-value < 0.00001. This indicates that the five most relevant features from the CNN are more relevant to a phenotype than the five most relevant features from a cTAKES-based model. The free-text comments confirm our descriptive results; the CNN-based phrases are seen as specific and directly relating to a patient’s condition while the CUI’s are seen as more generic. Moreover, clinicians were impressed to see phrases such as “h o withdrawal” for alcohol abuse (short for “history of”, which are typically difficult to interpret by non-experts. Some of the longer phrases from the CNN for the depression phenotype showed the word “depression” amids other diseases, indicating that the phrase is taken from a diagnosis section of the discharge summary. Clinicians commented that this helped them to contextualize the phrase and that it was more helpful than seeing the word “depression” in isolation. This indicates that giving further contextual information can help to increase understanding of predictions.

## Discussion

Our results show that CNNs provide a valid alternative approach to the identification of patient conditions from text. However, we notice a strong variation in the results between phenotypes with AUCs between 73 and 100, and F1-scores between 57 and 97, even with consistent annotation schemes. Some concepts such as Chronic Pain are especially challenging to detect, even with 321 positive examples in the data set. This makes it difficult to compare our results to other reported metrics in the literature, since studies typically consider different concepts for detection. This problem is further amplified by the sparsity of available studies that investigate unstructured data [13], and the lack of standardized datasets for this task. We hope that the release of our annotations will support work towards a more comparable performance in text-based phenotyping.

### Interpretability

Since bias in data collection and analysis is at times unavoidable, models are required to be interpretable in order for clinicians to be able to detect such biases, and alter the model accordingly [27]. Furthermore, interpretable models lead to an increased trust from the people who use them [26]. The interpretability is typically considered to be a major advantage of rule or concept-extraction based models that are specifically tailored to a given problem. Clinicians have full control over the dictated phrases that are used as input to a model. We demonstrated that CNNs can be interpreted in the same way as concept-extraction based models by computing the saliency of inputs. This even leads to a higher level of interpretability in that the extracted phrases are more relevant to a phenotype than the extracted CUIs. However, a disadvantage of CNNs is that they -by design- consider more different phrases than concept-extraction based methods. Thus, lists of salient phrases will naturally contain more items, making it more difficult to investigate which phrases lead to a prediction. However, restricting the list to a small number of items with the highest saliency coefficients or only including those above a saliency threshold can compensate for the length of the list. The question that developers of such models will ultimately have to answer is whether the trade-off between increased performance is worth the additional effort to extract and show relevant phrases.

### Alternative approaches

We further note that both concept-extraction and deep learning are supervised approaches and thus require a labeled dataset. The extraction of all labels for a single discharge summary took clinicians up to 10 minutes, which in our case (with twice-labeled notes) amounts to over 500 hours of annotation work across all annotating clinicians. Therefore, going forward it will be important to combine our approach with methods that alleviate this disadvantage by using semi-supervised methods that do not require fully labeled data-sets [16, 76, 77].

Another approach not considered here that does work without a large annotated data set is to develop a fully rule-based system. While a CNN learns the phrases that lead to a positive label, rule-based approaches require clinicians to define every phrase that is associated with a concept and establish links between them. An example by Mosley et al. looks for certain drug names and the word “cough” within the same line in a description of a patients’ allergies [78]. However, due to the heterogeneity of text, clinicians may be unable to consider all of the possible phrases in advance. They also have to consider how to handle negated phrases correctly. Finally, for some clinically important phenotypes such as “Non-Adherence”, it is impossible to construct an exhaustive list of associated phrases. Moreover, while rule-based systems require a separate algorithm for each concept, approaches that do not require concept-specific input such as CNNs can be trained for all phenotypes at the same time. This offers an opportunity to dramatically accelerate the development of scalable phenotyping algorithms for complex clinical concepts in unstructured clinical text that are poorly captured in the structured data. For example, being able to identify patients who are readmitted to hospital due to poor management of problems will have high clinical impact.

### Future extensions

While we consider and analyze a simple CNN architecture in this work, future extensions grounded in our findings could explore other deep learning model architectures. Alternatively, one could imagine different feature maps of a CNN for each of the sections in a medical record to capture additional information. Recurrent neural networks could also be applied to directly capture these context specific information instead of using convolutional neural networks. Finally, our unstructured data could be combined with structured input, such as lab results, data from UMLS, or ICD-codes. Augmenting unstructured text with structured data will help to achieve the best possible performance in a given phenotyping task [13].

We anticipate validation of our proposed approach in other types of clinical notes such as social work assessment to identify patients at risk for hospital admissions. Lastly, the CNN creates the opportunity to develop a model that can use phrase saliency to highlight notes and tag patients to support chart review. Future work will explore whether the identification of salient phrases can be used to support chart abstraction and whether models using these phrases represent what clinicians find salient in a medical note. Another opportunity that the learned phrases from a CNN provide is to better understand how different medical conditions are typically described in text. This knowledge can then be turned into improvements to systems like cTAKES and to build better predictive models with a human in the loop [79].

### Conclusion

Taking all these points into consideration, we conclude that deep learning provides a valid alternative to concept extraction based methods for patient phenotyping. Using CNNs can significantly improve the accuracy of patient phenotyping without needing any phrase-dictionary as input. We showed that concerns about the interpretability of deep learning can be addressed by computing a gradient-based saliency to identify phrases associated with different phenotypes. We propose that CNNs should be employed alongside concept-extraction based methods to analyze and mine unstructured clinical narratives and augment the structured data in secondary analysis of health records. The deep learning approach presented in this paper could further be used to assist clinicians during chart review by highlighting phrases related to phenotypes of a patient. Moreover, the same methodology could be used to support the identification of billing codes from text.

## Supporting information

S1 Table Overview of CNN results with different convolution widths. Each column name shows the minimum and maximum width of the convolution.
(PDF)

S2 Table Overview of cTAKES results with different models with all input features and the filtered lists of inputs. While in most cases, the clinician-defined phrase dictionary improves the model performance, the full input performs almost as well and outperforms the filtered model in some.
(PDF)

S3 Table Results of different n-gram based models. Each column name shows the minimum and maximum length of phrase that has been considered. We observe that in most cases, a simple bag of words (phrase length 1) outperforms all other models.
(PDF)

S1 Text Additional training information for the models. (PDF)
