# Supplementary material for: Comparing deep learning and concept extraction based methods for patient phenotyping from clinical narratives
Source: PLoS One. 2018 Feb 15;13(2):e0192360. doi: 10.1371/journal.pone.0192360 (PMC5813927; doi:10.1371/journal.pone.0192360)
Supplement: S1 Table — Each column name shows the minimum and maximum width of the convolution. (PDF) [file pone.0192360.s001.pdf]

Overview of CNN results with different convolution widths. Each column name shows the minimum and maximum width of the convolution.

		**1**	**1-2**	**1-3**	**1-4**	**1-5**	**2-5**
Adv. Cancer	P	62	**95**	74	87	77	90
	R	42	58	**65**	**65**	**65**	61
	$F1$	50	72	69	**74**	70	73
	AUC	85	94	93	**95**	93	93
Adv. Heart Disease	P	48	67	71	**74**	**74**	73
	R	71	64	66	63	**76**	68
	$F1$	58	66	68	68	**75**	70
	AUC	86	90	**92**	91	91	**92**
Adv. Lung Disease	P	27	46	50	39	37	**67**
	R	46	39	46	61	**68**	57
	$F1$	34	42	48	47	48	**62**
	AUC	83	89	**90**	**90**	**90**	89
Chronic Neuro	P	37	71	67	69	64	**81**
	R	68	45	61	**70**	68	61
	$F1$	48	55	63	**69**	66	**69**
	AUC	74	80	80	**84**	**84**	83
Chronic Pain	P	32	76	49	57	69	**78**
	R	**55**	39	**55**	43	32	45
	$F1$	41	52	52	49	44	**57**
	AUC	72	**78**	75	71	73	73
Alcohol Abuse	P	37	83	81	**86**	84	85
	R	57	68	**79**	68	75	**79**
	$F1$	45	75	80	76	79	**81**
	AUC	80	89	94	95	95	**96**
Substance Abuse	P	73	**84**	83	67	76	83
	R	27	70	83	**87**	83	80
	$F1$	39	76	**83**	75	79	81
	AUC	87	97	97	97	**98**	**98**
Obesity	P	17	95	95	**100**	95	**100**
	R	50	**95**	**95**	**95**	**95**	**95**
	$F1$	25	95	95	**97**	95	**97**
	AUC	69	**100**	**100**	**100**	**100**	**100**
Psychiatric Disorders	P	43	88	87	87	**90**	87
	R	54	76	78	**80**	76	**80**
	$F1$	48	82	82	**83**	**83**	**83**
	AUC	77	**95**	93	**95**	93	94
Depression	P	47	88	85	87	90	**91**
	R	44	76	79	**81**	79	76
	$F1$	45	81	82	**84**	**84**	83
	AUC	71	92	**93**	92	**93**	**93**
