# Supplementary material for: Comparing deep learning and concept extraction based methods for patient phenotyping from clinical narratives
Source: PLoS One. 2018 Feb 15;13(2):e0192360. doi: 10.1371/journal.pone.0192360 (PMC5813927; doi:10.1371/journal.pone.0192360)
Supplement: S2 Table — While in most cases, the clinician-defined phrase dictionary improves the model performance, the full input performs almost as well and outperforms the filtered model in some. (PDF) [file pone.0192360.s002.pdf]

Comparison between different cTAKES-based models with full and hand-filtered input.

		LR full	RF full	NB full	LR filter	RF filter	NB filter
Adv. Cancer	P	**86**	71	80	85	63	85
	R	58	48	**65**	55	39	55
	$F1$	69	58	**71**	67	48	67
	AUC	**94**	91	**94**	91	92	92
Adv. Heart Disease	P	**71**	62	52	73	59	**71**
	R	49	36	47	**59**	37	34
	$F1$	58	45	50	**65**	46	46
	AUC	88	88	83	**89**	88	87
Adv. Lung Disease	P	58	50	67	43	53	**100**
	R	25	14	29	**36**	29	14
	$F1$	35	22	**40**	39	37	25
	AUC	86	82	81	**87**	85	81
Chronic Neuro	P	75	**81**	64	80	73	78
	R	55	46	33	**62**	47	37
	$F1$	64	59	43	**70**	58	50
	AUC	**87**	86	77	86	**87**	84
Chronic Pain	P	49	66	36	58	66	**82**
	R	45	41	21	**52**	48	32
	$F1$	47	51	27	55	**56**	46
	AUC	78	78	73	83	**85**	81
Alcohol Abuse	P	88	67	63	62	**91**	73
	R	**79**	71	68	71	75	68
	$F1$	**83**	69	66	67	82	70
	AUC	95	91	91	94	**96**	93
Substance Abuse	P	**93**	81	67	86	89	87
	R	47	57	53	63	57	**67**
	$F1$	62	67	59	73	69	**75**
	AUC	97	92	95	97	95	**97**
Obesity	P	67	64	50	68	62	**100**
	R	70	**80**	15	65	75	35
	$F1$	68	**71**	23	67	68	52
	AUC	98	**99**	85	98	98	93
Psychiatric Disorders	P	70	74	67	78	**79**	81
	R	56	63	37	59	46	**64**
	$F1$	62	68	48	67	58	**72**
	AUC	87	88	80	90	88	**93**
Depression	P	81	76	74	78	79	**82**
	R	72	69	46	64	**77**	35
	$F1$	76	72	57	70	**78**	49
	AUC	**94**	91	80	90	91	85
