# Supplementary material for: Comparing deep learning and concept extraction based methods for patient phenotyping from clinical narratives
Source: PLoS One. 2018 Feb 15;13(2):e0192360. doi: 10.1371/journal.pone.0192360 (PMC5813927; doi:10.1371/journal.pone.0192360)
Supplement: S3 Table — Each column name shows the minimum and maximum length of phrase that has been considered. We observe that in most cases, a simple bag of words (phrase length 1) outperforms all other models. (PDF) [file pone.0192360.s003.pdf]

Overview of n-gram models with different phrase lengths. Each column name shows the minimum and maximum width of considered phrases. Phrase-length 1 is equivalent to a bag-of-words model.

		**1**	**1-2**	**1-3**	**1-4**	**1-5**
Adv. Cancer	P	**44**	41	41	41	35
	R	**77**	55	55	55	48
	$F1$	**56**	47	47	47	41
	AUC	**90**	88	87	86	85
Adv. Heart Disease	P	70	73	73	**78**	**78**
	R	32	27	32	36	**42**
	$F1$	44	40	45	49	**55**
	AUC	85	**86**	**86**	**86**	85
Adv. Lung Disease	P	21	**27**	**27**	23	21
	R	29	**39**	32	36	32
	$F1$	24	**32**	30	28	25
	AUC	76	**79**	78	78	77
Chronic Neuro	P	47	48	**49**	40	42
	R	46	53	54	**63**	62
	$F1$	46	50	**51**	49	50
	AUC	**72**	71	71	71	70
Chronic Pain	P	33	37	**42**	39	**42**
	R	**54**	39	38	46	46
	$F1$	41	38	40	43	**44**
	AUC	**68**	67	67	67	67
Alcohol Abuse	P	**100**	55	57	53	52
	R	50	**64**	57	57	57
	$F1$	**67**	59	57	55	54
	AUC	**89**	88	87	86	85
Substance Abuse	P	62	75	71	77	**83**
	R	**50**	30	33	33	33
	$F1$	**56**	43	45	47	48
	AUC	**90**	89	88	87	86
Obesity	P	27	16	40	42	**44**
	R	35	**45**	20	20	20
	$F1$	**30**	24	27	27	28
	AUC	**72**	**72**	**72**	**72**	71
Psychiatric Disorders	P	47	53	**55**	**55**	**55**
	R	**53**	39	31	29	31
	$F1$	**50**	45	39	38	39
	AUC	**77**	76	75	74	73
Depression	P	51	47	51	51	**52**
	R	67	**78**	73	73	71
	$F1$	58	59	**60**	**60**	**60**
	AUC	77	**78**	**78**	**78**	**78**
