# Supplementary material for: Comparing deep learning and concept extraction based methods for patient phenotyping from clinical narratives
Source: PLoS One. 2018 Feb 15;13(2):e0192360. doi: 10.1371/journal.pone.0192360 (PMC5813927; doi:10.1371/journal.pone.0192360)
Supplement: S1 Text — (PDF) [file pone.0192360.s004.pdf]

CNN Hyperparameters. We set the number of feature maps for each convolution width to 100 and used dropout with a probability of 0.5 to prevent overfitting [1]. All parameters were initialized using a uniform distribution from -0.05 to 0.05. The model was trained with adadelta [2]. After every parameter update, the parameters of the feature maps were normalized to a norm of 3.

Word Embedding Hyperparameters. We used word2vec to train the word embeddings on all the notes of MIMIC III v3. We used the continuous bag of words (CBOW) model, set the window size to 10, sampled 10 negative examples for each positive example, discarded words that appear less than 5 times, and performed 15 training iterations.

cTAKES Hyperparameters. We used cTAKES 3.2.2 with the default FilesInDirectoryCollectionReader.xml.

References

1. Srivastava N, Hinton G, Krizhevsky A, Sutskever I, Salakhutdinov R. Dropout: A simple way to prevent neural networks from overfitting. The Journal of Machine Learning Research. 2014;15(1):1929–1958.

2. Zeiler MD. ADADELTA: an adaptive learning rate method. arXiv preprint arXiv:12125701. 2012;.
